# Quantifying Cerebellum Grey Matter and White Matter Perfusion Using Pulsed Arterial Spin Labeling

**DOI:** 10.1155/2014/108691

**Published:** 2014-05-15

**Authors:** Xiufeng Li, Subhendra N. Sarkar, David E. Purdy, Richard W. Briggs

**Affiliations:** ^1^Department of Radiology and Center for Magnetic Resonance Research, University of Minnesota, 2021 Sixth Street SE, Minneapolis, MN 55455, USA; ^2^Department of Radiology, Beth Israel Deaconess Medical Center, Harvard Medical School, Boston, MA 02215, USA; ^3^Siemens Healthcare, Malvern, PA 19355, USA; ^4^Department of Radiology, UT Southwestern Medical Center, Dallas, TX 75390, USA; ^5^Department of Internal Medicine, UT Southwestern Medical Center, Dallas, TX 75390, USA; ^6^Department of Physics & Astronomy, Georgia State University, Atlanta, GA 30302, USA

## Abstract

To facilitate quantification of cerebellum cerebral blood flow (CBF), studies were performed to systematically optimize arterial spin labeling (ASL) parameters for measuring cerebellum perfusion, segment cerebellum to obtain separate CBF values for grey matter (GM) and white matter (WM), and compare FAIR ASST to PICORE. Cerebellum GM and WM CBF were measured with optimized ASL parameters using FAIR ASST and PICORE in five subjects. Influence of volume averaging in voxels on cerebellar grey and white matter boundaries was minimized by high-probability threshold masks. Cerebellar CBF values determined by FAIR ASST were 43.8 ± 5.1 mL/100 g/min for GM and 27.6 ± 4.5 mL/100 g/min for WM. Quantitative perfusion studies indicated that CBF in cerebellum GM is 1.6 times greater than that in cerebellum WM. Compared to PICORE, FAIR ASST produced similar CBF estimations but less subtraction error and lower temporal, spatial, and intersubject variability. These are important advantages for detecting group and/or condition differences in CBF values.

## 1. Introduction

The cerebellum is important for motor control, attention, language, and emotion [1, 2] and is compromised in a number of diseases, such as ataxia, autism, and cerebellar cognitive affective syndrome [3, 4]. Adequate perfusion of the tissue bed is important physiologically for tissue viability and function. Therefore, reliable assessments of cerebellum perfusion are valuable for studying the normal physiology of the cerebellum, diagnosing and providing information about pathology, and monitoring the efficacy of individualized therapy strategies [5, 6].

As a completely noninvasive perfusion imaging technique, arterial spin labeling (ASL) has been useful for both clinical [7–10] and research [11–13] studies. However, most of the technical developments and applications of ASL have focused on the superior cortical part of the brain rather than inferior brain regions such as cerebellum. In addition to two abstracts with preliminary data from optimization experiments [14, 15] and a case study [16], only a handful of papers have reported cerebellum CBF values [17–22], and only one has reported separate CBF values for grey matter (GM) and white matter (WM) in cerebellum [17].

FAIR ASST [15, 17], by using preinversion and postinversion superior saturations, overcomes the FAIR confound of creating two labeled boluses, only one of which is temporally defined with QUIPSS [13] or Q2TIPS [23, 24]. FAIR ASST also reduces adverse venous artifacts generated by the intrinsic superior labeling of traditional FAIR when mapping CBF in middle and inferior brain regions. In this respect, FAIR ASST is similar to asymmetric PASL techniques such as PICORE (proximal inversion of magnetization with a control for off-resonance effects) [13], in which only the inferiorly labeled blood is used for perfusion quantification; yet FAIR ASST retains the inherent control of magnetization transfer (MT) effects of symmetric FAIR methods. The aims of this study were to (1) systematically optimize ASL parameters for quantifying cerebellum CBF, (2) segment cerebellum to obtain separate CBF values for grey matter (GM) and white matter (WM), and (3) verify the hypothesis that FAIR ASST would be better than PICORE for quantifying cerebellum perfusion.

## 2. Materials and Methods

### 2.1. Subjects

Ten healthy male adults (age range 25–37 years, mean ± S.D. = 29 ± 6 years) took part in four studies in three sessions to optimize the ASL sequences for the cerebellum: three subjects for the first study and session, four for the second study and session, and another three for the last two studies in the third session (see “ASL Optimization Studies” below). Five healthy male adults (age range 27–40 years, mean ± S.D. = 35 ± 5 years) participated in the subsequent quantitative cerebellum perfusion study. Since caffeine is a vasoconstrictor, all subjects refrained from caffeine-containing coffee, tea, and soft drinks at least 8 hours before the studies. Subjects were instructed to avoid any movements of arms or legs and keep eyes closed but remain awake. Subjects' heads were tightly restrained with foam padding. Studies were performed at approximately the same time in the early afternoon to avoid potential effects of circadian rhythms. Informed consent was obtained from all subjects prior to the studies, according to a protocol approved by the local Institutional Review Board.

### 2.2. MRI Scanner and Sequences

Studies were performed using a 3T Siemens Trio TIM whole-body scanner with 60 cm diameter magnet bore and SQ gradients (maximum gradient strength 45 mT/m in the *z* direction and 40 mT/m in the *x* and *y* directions, maximum slew rate 200 T/m/s). The body coil was used for transmission, and the Siemens 12-channel phased array head coil was used for reception.

**Figure 1:**
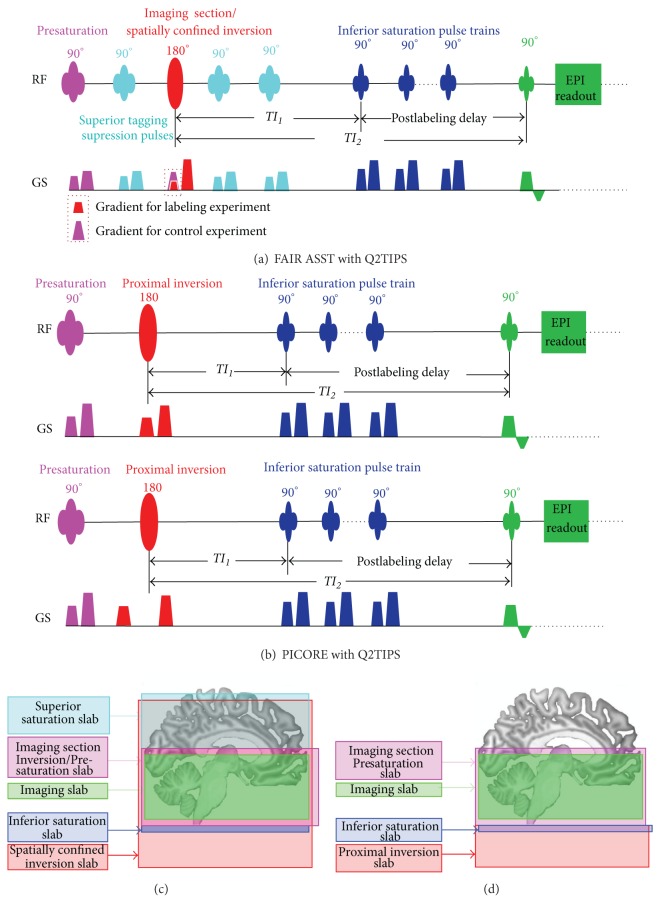
Sequence diagrams for FAIR ASST (a) and PICORE (b) and spatial definitions for different slabs of FAIR ASST (c) and PICORE (d). Imaging section presaturation pulses (a, b in pink) were played out before the inversion RF pulses (in red); the imaging section slabs for the presaturations are presented in pink (c, d). Inferior saturation pulses and the corresponding saturation slab are indicated by dark blue. Superior tagging suppression pulses and the corresponding saturation slab, for FAIR ASST only, are displayed in cyan (a, c). For FAIR ASST (a, c), the stronger gradient (pink) for the imaging section control inversion and the weaker gradient (red) for the spatially confined labeling inversion are superposed on the sequence diagram and further denoted by the legends beneath the sequence diagram. For PICORE (b, d), the labeling was achieved by using proximal inversion (red) 10 mm below the imaging slab (green); in the labeling experiment (b, top), the slice-selective gradient was played out with an RF inversion pulse to create the proximal inversion slab (d), while in the control experiment (b, bottom), the gradient was played out before the control off-resonance RF inversion pulse, to control for possible gradient eddy current effects. The imaging slab was positioned to make the first inferior imaging slice cover the inferior edge of the cerebellum (c, d).

For both FAIR ASST and PICORE sequences (Figure 1), a 15.36 ms hyperbolic secant inversion pulse with 22 *μ*T RF amplitude and 95% labeling efficiency was used. The slab thickness for the inversion was adjusted by varying the slice-select gradient amplitude based on a reference value of 0.7 mT/m for a 100 mm slab [25]. Both sequences incorporated Q2TIPS method to define temporal bolus duration (*TI*_1_) [24]. After a postlabeling time, imaging slices were acquired at total delay time (*TI*_2_) by using echo planar imaging (EPI) as the readout. All saturation modules consisted of a 90-degree RF pulse followed by a spoiling gradient. The total duration of one saturation module was about 11 ms. Depending on the thickness of the saturation slab, an appropriate RF saturation pulse was automatically selected from a set of sinc RF pulses optimized for various thicknesses.

The FAIR ASST [15, 17] sequence used one preinversion and two postinversion superior saturation RF pulses to suppress the superior tagging of FAIR. The PICORE sequence performed labeling image acquisition with proximal inversion and control image acquisition without slice-selective proximal inversion. Instead, the gradient used for slice-selective inversion in the labeling image acquisition was played out before the frequency-offset RF pulse [13], in the control image acquisition (Figure 1), to control for possible eddy current effects on slice acquisition.

### 2.3. Imaging Protocol

The Siemens coregistration tool AutoAlign was used to provide consistent slice orientation and position with respect to a standard head atlas across subjects and sessions. Axial ASL imaging slices were positioned to have the first inferior slice cover the lower edge of the cerebellum, oriented parallel to the anterior-posterior direction (Figure 1). Due to subject-dependent brain geometry, manual translation adjustment was helpful to ensure consistent slice position across subjects. The AutoAlign scout was followed by a gradient echo localizer, the *T*_1_-weighted high-resolution anatomic imaging sequence MPRAGE, and ASL scans with an EPI readout.

The MPRAGE sequence used the following parameters: TR/TE/TI = 2250/4/900 ms, flip angle = 9°, field of view (FOV) = 230 × 230 × 160 mm^3^, matrix size = 256 × 256 × 160, resolution = 0.9 × 0.9 × 1 mm^3^, bandwidth = 160 Hz/pixel, GRAPPA iPAT factor = 2 with 24 reference lines, partial Fourier (PF) = 7/8, slice oversampling = 10%, slice orientation = sagittal, phase encoding direction = anterior to posterior, and total imaging time = 4 minutes and 38 seconds.

### 2.4. ASL Optimization Studies

**Figure 2:**
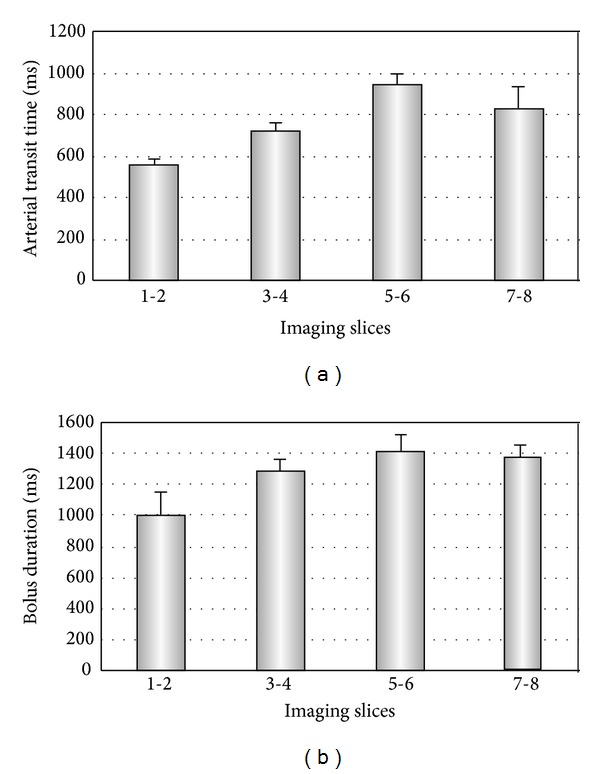
Estimated arterial transit time (a) and bolus duration (b) from perfusion studies using varied total delay times, *TI*_2_. Error bars represent standard deviations.

The following ASL studies were performed to optimize FAIR ASST parameters for measuring cerebellar CBF: (1) a series of perfusion experiments, each with a different *TI*_2_ value, to estimate arterial transit time (ATT) and bolus duration, the latter for properly setting the *TI*_1_ value for the following experiments (Figure 1), (2) a perfusion study using the fixed optimal *TI*_1_ value of 800 ms determined in (1) and varied postlabeling delay times to identify the proper delay to avoid intravascular artifacts, (3) a multiple-TR perfusion study to determine if a short TR significantly affects the refreshment of arterial blood at the labeling site, and (4) a study varying the number of inferior saturation pulses to find the minimum number necessary for effective suppression of residual labeled blood spins.

The variable *TI*_2_ perfusion study employed the following MRI parameters: TR/TE = 4000/9.2 ms, FOV = 230 × 230 mm^2^, matrix size = 66 × 66, in-plane resolution = 3.48 × 3.48 mm^2^, number of imaging slices = 12, slice thickness/gap = 5 mm/1 mm, imaging slab size = 71 mm, pairs of label and control measurements = 30, iPAT GRAPPA factor = 2 with 24 reference lines, and partial Fourier (PF) = 7/8. Imaging slices were acquired in ascending order. The slab sizes for the superior saturation, imaging section inversion, and spatially confined selective inversion were equal to 100 mm, 91 mm, and 271 mm, respectively. For each subject, the experiment was conducted twelve times using randomly ordered *TI*_2_, values of 50, 300, 600, 900, 1200, 1500, 1800, 2100, 2400, 2700, 3000, and 3300 ms. The inferior saturation pulse train was turned off. Total imaging time for ASL acquisitions was about 48 minutes.

The rest of the optimization studies used the same ASL parameters as those in the studies with varied *TI*_2_ values, except as follows. Parameters for perfusion studies using varied postlabeling delay times were TR = 3000 ms, inferior saturation pulse interval/thickness = 25 ms/20 mm, *TI*_1_ value/postlabeling times = 800/{200,400,600,800,1000,1200,1400} ms (randomized for each subject), inferior saturation pulse train lasting for the entire postlabeling delay period, and total ASL imaging time of about 21 minutes. In the perfusion studies exploring the effect of TR, parameters were TR = {2.5,3.0,3.5} s (randomized for each subject), *TI*_1_/*TI*_2_ = 800 ms/1800 ms, inferior saturation pulse interval/thickness/number = 25 ms/20 mm/40, and total imaging time of about 9 minutes. In the study evaluating the number of inferior saturations, parameters were TR = 3 s, *TI*_1_/*TI*_2_ = 800 ms/1800 ms, and inferior saturation pulse interval/thickness = 25 ms/20 mm. The number of inferior saturation pulses was varied from 0 to 40 in increments of 10, and the order of the imaging scans was randomized for each subject. The total imaging time for evaluating the sufficient number of inferior saturations was about 15 minutes.

### 2.5. Quantitative Cerebellum Perfusion Studies

The following ASL parameters were used for quantitative cerebellum perfusion studies: FOV = 180 × 180 mm^2^, matrix size = 72 × 72, slice thickness/gap = 3.5/0.7 mm, slice number = 16, in-plane resolution = 2.5 × 2.5 mm^2^, slice thickness/gap = 3.5/0.7 mm, TR/TE = 2500/12 ms, pairs of label and control measurements = 90, iPAT GRAPPA factor = 2 with 24 reference lines, partial Fourier (PF) = 7/8, acquisition order = ascending (foot to head), *TI*_1_/*TI*_2_ = 800 ms/1800 ms, inferior saturation interval/thickness/number = 25 ms/20 mm/20, superior saturation slab thickness = 100 mm, selective inversion slab = 86.5 mm, and spatially confined inversion slab = 266.5 mm. For PICORE, a 100 mm slab, approximately the same as the effective inferior labeling slab size applied in FAIR ASST experiments, was used for proximal inversion, with a 10 mm gap between the imaging slab and the inversion slab. For each ASL scan, two proton density (*M*_0_) images were acquired, one before and one after each ASL series, by using the same sequence with TR = 8 s, and the average *M*_0_ image was used in subsequent model fits [25]. The total ASL imaging scan time was about 16 minutes with about 8 minutes for each method.

### 2.6. Data Processing Software

Image processing operations, such as motion correction and coregistration, were performed with SPM2 (Functional Imaging Laboratory, University College London). Four-parameter iterative nonlinear least squares model fitting was performed using scripts implemented in Matlab 7.1 (The MathWorks, Inc., Natick, Massachusetts) for perfusion signals from studies using multiple varied *TI*_2_ values.

### 2.7. Image Preprocessing

Each ASL imaging series was first evaluated for subject motion, and whenever the translational motion was larger than 1 mm or the rotation around any axis was larger than 1°, motion correction was performed using trilinear interpolation. Pairs of labeling and control images with motions larger than 2 mm in translation or 2° in rotation around any axis were excluded from further processing or analysis. A mean image of the ASL series was generated for later use in coregistration. Each ASL label-control image series was processed by pairwise subtraction to generate a perfusion-weighted imaging series, and images within the series were averaged to produce a mean perfusion-weighted image. The two *M*_0_ images in each series were averaged to obtain a mean *M*_0_ image.

### 2.8. Iterative Model Fitting

An iterative nonlinear least-squares model fitting was performed to fit ASL signals in the defined ROIs to the three-phase, single blood compartment model [26]:

(1) ΔM(t)=0, 0<t<Δt=2αM0bCBF(t−Δt)exp⁡⁡(−tT1b), Δt<t<τ+Δt=2αM0bCBFτexp⁡⁡(−tT1b), τ+Δt  <t,

where Δ*M*(*t*) is the measured ASL difference signal between label and control images in a specified ROI or voxel at inversion time *t*, Δ*t* is the arterial transit time, *τ* is the bolus duration, CBF is the cerebral blood flow, *M*_0*b*_ is the fully relaxed magnetization of the blood, *T*_1*b*_ is the longitudinal relaxation time of the arterial blood, and *α* is the labeling efficiency, assumed to be 0.95 for the hyperbolic secant pulse used in this study [25].

To avoid the adverse effects of labeled blood signals from large arteries at short postlabeling delay times and of subtraction errors due to small motions, trimmed means of averaged ASL difference signals from the studies using varied *TI*_2_ values were used for ASL model fitting, by excluding the 5% of voxels with the lowest values and the 5% with the highest values within GM and WM ROIs [27]. Perfusion signals were compensated for longitudinal relaxation differences between slices due to the incremental time of ~30 ms for sequential slice acquisition. To increase the signal-to-noise ratio (SNR) for perfusion signals measured with long *TI*_2_ values, the GM perfusion signals of two adjacent imaging slices were averaged and fitted to the perfusion model.

### 2.9. CBF Quantification Using the Single-Subtraction Method

The mean perfusion-weighted image and the mean *M*_0_ image were used to estimate CBF using the single blood compartment model [25, 28, 29]:

(2) $$\begin{array}{c} \text{CBF}=\frac{\Delta M}{\left(2\alpha M_{0b}{TI}_{1}\exp\left(-TI_{2}/T_{1b}\right)\right)}, \end{array}$$

(3) $$\begin{array}{c} M_{0b}=\frac{M_{0}}{\lambda }, \end{array}$$

where Δ*M* is the mean ASL difference signal between labeling and control images, *TI*_2_ is the total inversion time equal to the sum of *TI*_1_ and the postlabeling delay, *M*_0_ is the measured tissue proton density, and *λ* is the brain blood/tissue partition coefficient (assumed to be 0.9) [30].

### 2.10. Segmentation of Cerebellum GM and WM

Individual whole-brain GM and WM tissue masks were automatically generated using SPM with a probability threshold of 0.75. To obtain GM and WM masks constrained to cerebellum, a mask for cerebellum was manually traced from the high-resolution anatomic image for each subject, and a Boolean operation was then performed between the hand-drawn cerebellar ROIs and the probability-based GM and WM segmentation maps from SPM. GM and WM segmentation masks constrained to cerebellum obtained from that operation were coregistered to the mean image of the ASL scan series. The cerebellar GM and WM masks occasionally exhibited some nonbinary voxel values due to interpolation, and a few of these intermediate-valued voxels extended slightly beyond the cerebellum. In these cases, a threshold (typically equal to 0.9 for most subjects) was used to further conservatively limit the masks, based on individual inspection.

### 2.11. ROI-Based Cerebellum GM and WM CBF Analysis

For each subject, overall cerebellum GM and WM CBF values were estimated for the two PASL methods. Group mean CBF and standard deviation were calculated for cerebellum GM and WM. Interslice, spatial, and temporal variabilities were evaluated by computing the coefficient of variance (C.V.), or standard deviation divided by mean, expressed as percentage. Interslice variability was defined as the ratio between the standard deviation and the mean of slice mean CBF values. Spatial variability was calculated for all voxels within cerebellum GM and WM. The temporal variability was calculated by using the mean perfusion signals of four imaging slices near transverse sinuses.

Comparisons of GM CBF, WM CBF and perfusion signal variability between the two PASL methods were performed using two-tailed paired *t*-tests; *P* < 0.05 was used as the threshold of statistically significant difference.

## 3. Results

### 3.1. Optimization Studies

Model-fitting the data from perfusion studies using varied *TI*_2_ values indicated that the estimated bolus durations (ca. 800–1500 ms) and arterial transit times (ca. 550–1000 ms) varied from slice to slice in the cerebellum (Figure 2). For the inferior saturation pulse to effectively define the labeled bolus, *TI*_1_ should be set less than the bolus duration *τ* [23]; a *TI*_1_ of 800 ms was chosen for subsequent optimization studies and quantitative cerebellum CBF estimation using FAIR ASST and PICORE.

**Figure 3:**
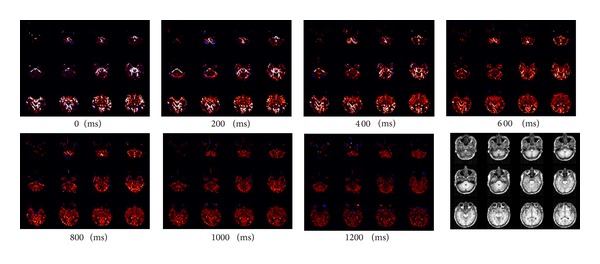
One typical subject's perfusion-weighted imaging maps and coregistered high-resolution anatomic images from the multiple-postlabeling-delay perfusion study with *TI*_1_ equal to 800 ms. Postlabeling delay times (ms) for perfusion-weighted imaging maps are presented under corresponding panels.

**Figure 4:**
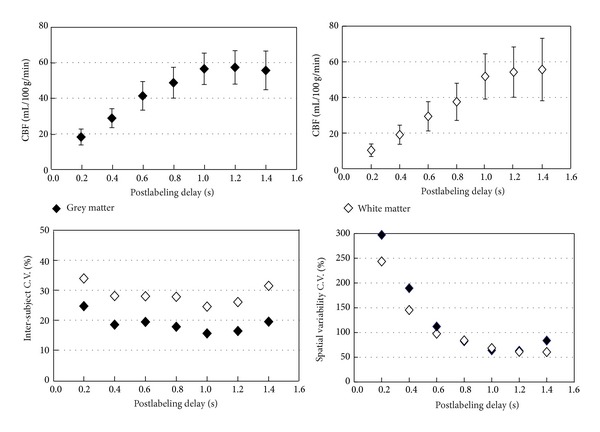
CBF values and intersubject and spatial variability of perfusion signals as a function of postlabeling delay time. Error bars represent standard deviation.

Typical perfusion-weighted imaging maps from studies using 800 ms *TI*_1_ and varied postlabeling delay times showed that at short delay times, spurious hyperintense signals within big arteries dominated, whereas after a delay of 1000 ms, the perfusion-weighted imaging maps became spatially uniform without obviously hyperintense signals. With a longer delay of 1200 ms, the perfusion-weighted imaging maps only marginally improved in signal uniformity while SNR decreased (Figure 3). Intersubject variability and spatial variability minimized at a postlabeling delay time of about 1000 ms, also implying that the optimal postlabeling delay time should be 1000 ms (Figure 4).

**Figure 5:**
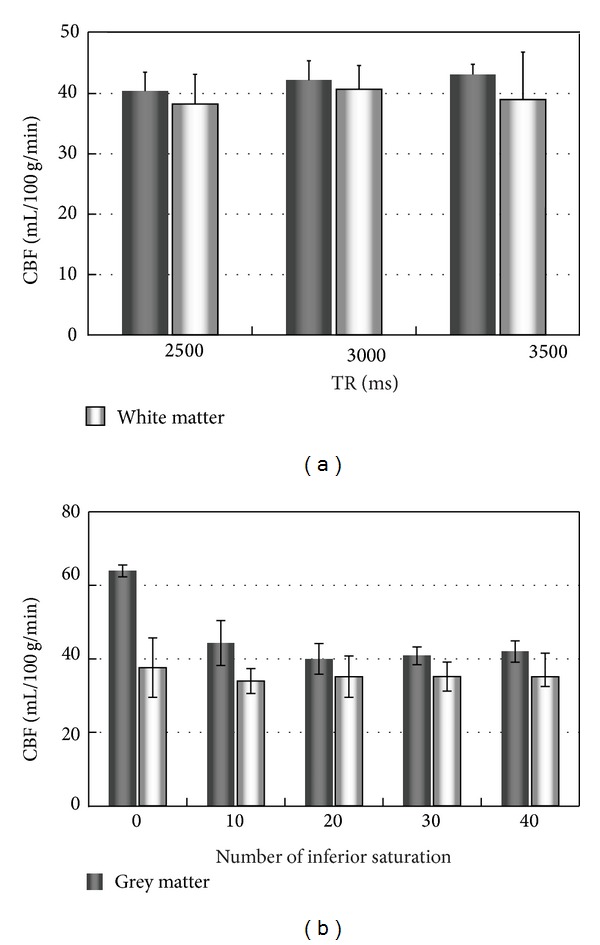
CBF values in cerebellum grey and white matter using different TRs (a) and different numbers of inferior saturation pulses (b). Error bars represent standard deviation.

CBF values measured using TR values of 2.5, 3.0, and 3.5 s were comparable for both GM and WM, indicating that blood at the labeling site is rapidly refreshed and that TR values as short as 2.5 s can be used for quantitative cerebellum perfusion studies at 3T (Figure 5(b)). Ten or more inferior saturation RF pulses were required for effective suppression; after 20 inferior saturation pulses, suppression remained almost unchanged when additional inferior saturation pulses were applied (Figure 5(a)).

### 3.2. Quantitative Perfusion Studies

**Figure 6:**
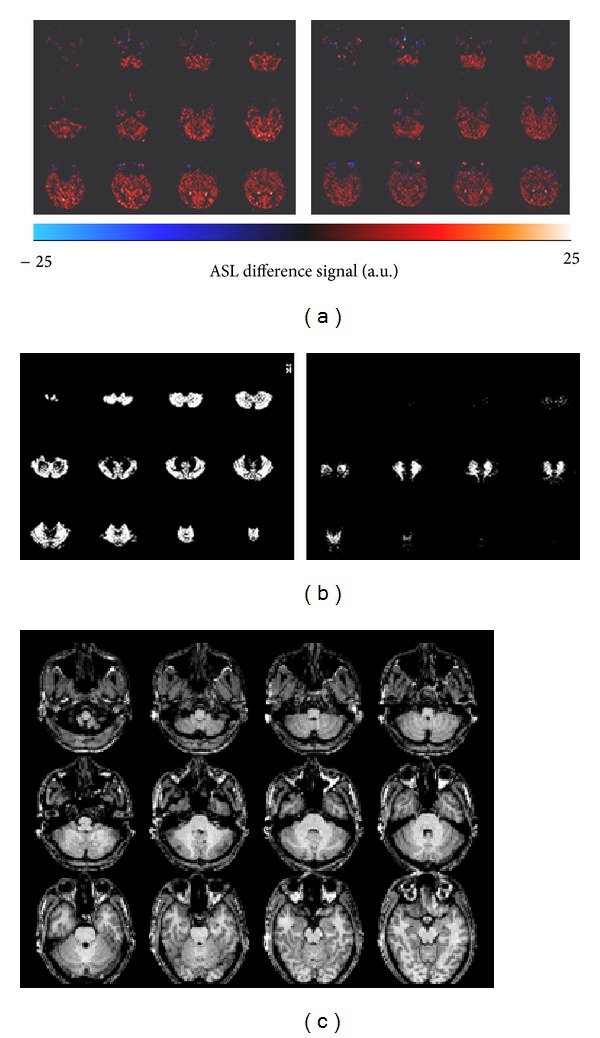
One typical subject's perfusion-weighted imaging maps in 12 slices from FAIR ASST ((a) left) and PICORE ((a) right), coregistered segmentation masks for cerebellum grey matter ((b) left) and white matter ((b) right), and corresponding high-resolution anatomic images (c).

Figure 6 shows, for a representative subject, a coregistered high-resolution anatomic image, the segmentation masks for cerebellum GM and WM, and the corresponding perfusion-weighted imaging maps from FAIR ASST and PICORE. Perfusion-weighted imaging maps from FAIR ASST have better uniformity than those from PICORE. The latter showed obvious hyperintense perfusion signal (especially in the inferior imaging slices) and more subtraction errors (as indicated by negative perfusion signals).

**Figure 7:**
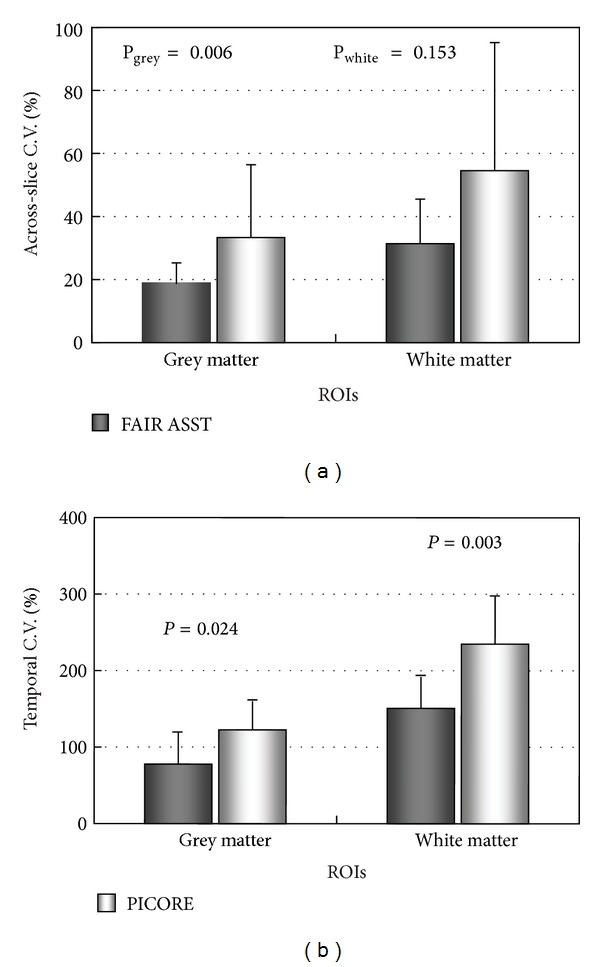
Across-slice and temporal variability of CBF values obtained with FAIR ASST and PICORE. Error bars represent standard deviations; *P* values are from two-tailed *t*-tests.

The mean CBF measured by FAIR ASST for cerebellum GM was 43.8 ± 5.1 mL/100 g/min, slightly higher but not significantly different (*P* = 0.135, paired two-tailed *t*-test) from the value of 40.35 ± 8.5 mL/100 g/min that was obtained using PICORE (Table 1). For cerebellum WM, the mean CBF from FAIR ASST was 27.6 ± 4.5 mL/100 g/min, not significantly different (*P* = 0.278, paired two-tailed *t*-test) from the slightly lower value of 23.7 ± 7.5 mL/100 g/min from PICORE (Table 1). Mean grey-to-white matter ratios of cerebellum CBF (Table 1) obtained using the two PASL methods are similar (*P* = 0.436, paired two-tailed *t*-test). CBF measured by FAIR ASST gave lower intersubject (Table 1) and interslice (Figure 7(a)) variability and were more stable with time than data from PICORE (Figure 7(b)).

### 3.3. Susceptibility and Volume Averaging Effects

**Figure 8:**
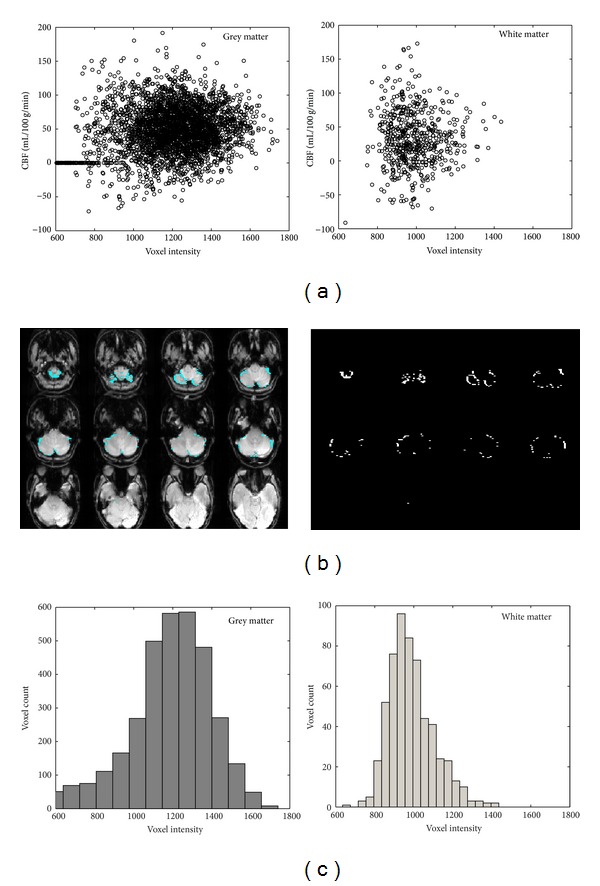
Analysis results for evaluating susceptibility and partial volume effects on cerebellar CBF measurements: (a) scatter plots of CBF versus *M*_0_ voxel intensity for cerebellar grey matter (left) and white matter (right) from one typical subject show no relation between these two parameters; (b) grey matter voxels with intensity <800 were located almost entirely at the superficial cerebellar boundaries, as shown by images with (left) and without (right) anatomic underlays; (c) histogram distribution plots of cerebellar grey matter and white matter *M*_0_ signal intensities from one typical subject showing high-intensity tail for WM and low-intensity tail for GM.

To check if signal reduction and image distortions arising from magnetic susceptibility gradients near the cerebellum [31] might be impacting CBF measurements of cerebellum GM or WM, scatter plots of CBF versus voxel intensity from the *M*_0_ images were made (Figure 8(a)) and Pearson correlation tests were run on the data. No systematic correlation of CBF and *M*_0_ voxel intensity was observed for WM (*R* ^2^ = 0.022); a slight correlation of CBF and *M*_0_ voxel intensity for GM (*R* ^2^ = 0.404) disappeared when the 9.6% of GM voxels with intensities less than 800 were ignored (*R* ^2^ = 0.029). The correlation of CBF and *M*_0_ voxel intensity for WM was only slightly affected by this threshold (1.6% of white matter voxels, *R* ^2^ = 0.018). The GM voxels with intensity < 800 were located almost entirely at the superficial cerebellar boundaries (Figure 8(b)), more suggestive of volume averaging (possibly exacerbated by slight motions) with the surroundings than of interfacial susceptibility mismatches. Histogram distribution plots for GM and WM *M*_0_ voxel intensities were slightly skewed to high intensity for WM and to low intensity for GM, suggesting some averaging of intensities on the grey-white matter boundary (Figure 8(c)).

## 4. Discussion

Reliable perfusion measurements depend upon the proper selection of ASL parameters [26, 32, 33]. Optimization of parameters for cerebellum perfusion studies at 3T indicated that a temporal bolus width of 800 ms and a postlabeling delay of 1000 ms were suitable, that TR could be as short as 2.5 s without degrading SNR, and that 20 inferior saturation pulses were sufficient to suppress the residual labeled arterial blood. The postlabeling delay of 1.0 s is the same as that selected in a recent optimization study of cerebellar CBF [14].

Several previous quantitative measurements of cerebellar CBF by PET [33], SPECT [34], and ASL [14, 21] have yielded values in the range of 58–65 mL/100 g/min. The cerebellum GM CBF values of 63.6 ± 5.0 mL/100 g/min estimated using a PCASL multiple inversion time experiment with 3 × 3 × 7 mm^3^ voxels [14] and of 58–62 mL/100 g/min estimated using PCASL with 3.44 × 3.44 × 5 mm^3^ voxels [21] agree well with the cerebellum GM CBF value of 56.7 ± 5.0 mL/100 g/min for 6 subjects using FAIR with Q2TIPS with (3.5 mm)^3^ voxels [17] and the cerebellum GM CBF values of 55–65 mL/100 g/min obtained in the inferior saturation and postlabeling delay experiments of this study. PET [34], SPECT [35–37], and other ASL studies, one with 7 × 3 × 3 mm^3^ voxels [20] and others with smaller voxels [16, 18, 19], have reported cerebellum CBF values of 30–48 mL/100 g/min, similar to those obtained with FAIR ASST in this study. The superior saturation of FAIR ASST reliably reduces contributions of inflow of superiorly labeled blood that artifactually enhance CBF estimates [15, 17] in FAIR, and thus it is not surprising that the cerebellar GM CBF of 43.8 ± 5.1 mL/100 g/min obtained using FAIR ASST with 2.5 × 2.5 × 3.5 mm^3^ voxels in this study is lower than those obtained using traditional FAIR [17]. GM CBF measurements by FAIR ASST are comparable to, although insignificantly and slightly lower than, the literature values from PCASL [14, 20–22]; these slight differences of CBF measurements between FAIR ASST and PCASL can possibly be attributed to the use of different ASL techniques as well as CBF quantification models [22]. The cerebellar WM CBF of 27.6 ± 4.5 mL/100 g/min from this FAIR ASST study is also lower than the cerebellar WM CBF of 36.7 ± 2.7 mL/100 g/min from FAIR with Q2TIPS [17]. It should be noted that our data indicate volume averaging of GM and WM in some voxels (Figure 8(c)); if this were corrected [38], our average GM CBF values would be somewhat greater and our average WM CBF values would be slightly smaller.

Although it has been suggested that EPI-based ASL sequences for obtaining cerebellar perfusion might exhibit signal reduction and image distortions arising from magnetic susceptibility gradients near the cerebellum [31], these problems were not observed in this study or others recently reported [14, 15, 17]. One reason might be that in these cases, parallel imaging was employed with short TE that reduces susceptibility effects in EPI.

These studies were performed on young healthy adults in the age range 25–40 years. For elderly subjects and patients, the blood velocity is lower [39, 40], which will require longer postlabeling delay to allow the labeled blood to wash out from larger arteries. More inferior saturation RF pulses may also have to be performed in elderly or ill subjects in order to completely suppress slow-moving labeled blood spins.

Because the superior inflow of venous blood is suppressed at the outset in FAIR ASST, confounding venous inflow effects are minimized, resulting in perfusion images with more stable ASL signals, as indicated quantitatively by the temporal stability analysis (Figure 4). The amount and velocity of superiorly labeled venous blood are subject dependent, which may account for why CBF measurements by FAIR ASST gave consistently lower intersubject variability than those by PICORE (Table 1). The observed smaller subtraction errors and about 50% lower interslice variability in perfusion images from FAIR ASST compared to PICORE (Figure 4) may be due to the better control of MT effects with FAIR than PICORE. Although we have not compared the two methods on patient groups, these results suggest that FAIR ASST will be more sensitive and specific than PICORE in detecting abnormal CBF values in patient groups and disease conditions.

## 5. Conclusions

Using properly selected ASL parameters based on the results of ASL optimization studies, CBF values for cerebellum GM and WM were measured using FAIR ASST and PICORE. Results indicated that FAIR ASST is preferable to PICORE, giving similar CBF estimations but with lower intersubject and spatial variability, less subtraction error, and greater temporal stability. These are important advantages, which should make FAIR ASST more sensitive and specific than PICORE in detecting abnormal CBF values in different conditions and/or subject groups.

## Figures and Tables

**Table 1:** Cerebellum CBF (mL/100 g/min) measured by FAIR ASST and PICORE^*∗*^.

Subject number	FAIR ASST			PICORE		
	CBF_GM_	CBF_WM_	CBF_GM_/CBF_WM_	CBF_GM_	CBF_WM_	CBF_GM_/CBF_WM_
1	42.58	29.56	1.44	41.97	33.5	1.25
2	45.07	28.63	1.57	44.47	28.89	1.54
3	37.89	20.48	1.85	30.28	19.04	1.59
4	41.82	26.62	1.57	33.59	14.71	2.28
5	51.57	32.53	1.59	51.45	22.51	2.29
Mean	43.78^*∗*^	27.56^*∗*^	1.60^*∗*^	40.35^*∗*^	23.73^*∗*^	1.79^*∗*^
S.D.	5.06	4.49	0.15	8.52	7.53	0.47
C.V. (%)	11.6%	16.3%	9.4%	21.1%	31.7%	26.3%

^*∗*^CBF_GM_ represents grey matter CBF; CBF_WM_: white matter CBF; C.V.: coefficient of variance; and S.D.: standard deviation. No significant differences were found between the mean cerebellum CBF values obtained with FAIR ASST and PICORE, but the C.V. for FAIR ASST is about half that for PICORE.
